# Imaging Protocol, Feasibility, and Reproducibility of Cardiovascular Phenotyping in a Large Tri-Ethnic Population-Based Study of Older People: The Southall and Brent Revisited (SABRE) Study

**DOI:** 10.3389/fcvm.2020.591946

**Published:** 2020-11-13

**Authors:** Lamia Al Saikhan, Muath Alobaida, Anish Bhuva, Nish Chaturvedi, John Heasman, Alun D. Hughes, Siana Jones, Sophie Eastwood, Charlotte Manisty, Katherine March, Arjun K. Ghosh, Jamil Mayet, Ayodipupo Oguntade, Therese Tillin, Suzanne Williams, Andrew Wright, Chloe Park

**Affiliations:** ^1^Department of Cardiac Technology, College of Applied Medical Sciences, Imam Abdulrahman Bin Faisal University, Dammam, Saudi Arabia; ^2^MRC Unit for Lifelong Health and Ageing, Department of Population Science & Experimental Medicine, UCL Institute of Cardiovascular Science, University College London, London, United Kingdom; ^3^Department of Basic Science, Prince Sultan Bin Abdulaziz College for Emergency Medical Services, King Saud University, Riyadh, Saudi Arabia; ^4^National Heart & Lung Institute, Imperial College London and Imperial College Healthcare NHS Trust, Hammersmith Hospital, London, United Kingdom; ^5^Cardio-Oncology Service, Department of Cardiology, Barts Heart Centre, Barts Health NHS Trust, St Bartholomew's Hospital, London, United Kingdom; ^6^Cardio-Oncology Service, Department of Cardiology, University College London Hospital, London, United Kingdom

**Keywords:** population-based, cardiovascular, imaging, echocardiography, vascular, feasibility, reproducibility

## Abstract

**Background:** People of South Asian and African Caribbean ethnicities living in UK have a high risk of cardiometabolic disease. Limited data exist regarding detailed cardiometabolic phenotyping in this population. Methods enabling this are widely available, but the practical aspects of undertaking such studies in large and diverse samples are seldom reported.

**Methods:** The Southall and Brent Revisited (SABRE) study is the UK's largest tri-ethnic longitudinal cohort. Over 1,400 surviving participants (58–85 years) attended the 2nd study visit (2008–2011); during which, comprehensive cardiovascular phenotyping, including 3D-echocardiography [3D-speckle-tracking (3D-STE)], computed tomography, coronary artery calcium scoring, pulse wave velocity, central blood pressure, carotid artery ultrasound, and retinal imaging, were performed. We describe the methods used with the aim of providing a guide to their feasibility and reproducibility in a large tri-ethnic population-based study of older people.

**Results:** Conventional echocardiography and all vascular measurements showed high feasibility (>90% analyzable of clinic attendees), but 3D-echocardiography (3DE) and 3D-STE were less feasible (71% 3DE acquisition feasibility and 38% 3D-STE feasibility of clinic attendees). 3D-STE feasibility differed by ethnicity, being lowest in South Asian participants and highest in African Caribbean participants (*p* < 0.0001). Similar trends were observed in men (*P* < 0.0001) and women (*P* = 0.005); however, in South Asians, there were more women with unreadable 3D-images compared to men (67 vs. 58%). Intra- and inter-observer variabilities were excellent for most of conventional and advanced echocardiographic measures. The test-retest reproducibility was good-excellent and fair-good for conventional and advanced echocardiographic measures, respectively, but lower than when re-reading the same images. All vascular measures demonstrated excellent or fair-good reproducibility.

**Conclusions:** We describe the feasibility and reproducibility of detailed cardiovascular phenotyping in an ethnically diverse population. The data collected will lead to a better understanding of why people of South Asian and African Caribbean ancestry are at elevated risk of cardiometabolic diseases.

## Introduction

People of South Asian and African Caribbean descent are known to experience an increased burden of diabetes and cardiovascular disease (CVD) compared with people of European ancestry; although why remains unclear (1). The Southall and Brent Revisited (SABRE) study was designed to address this question by establishing the relationships between risk factors, subclinical disease, and adverse clinical outcomes and mortality in a longitudinal cohort (2).

Methods enabling detailed cardiometabolic phenotyping are widely available, but the practicalities undertaking phenotyping in large and diverse study populations are seldom reported (3). We describe the clinical cardiovascular imaging acquisition and analysis methods performed on over 1,400 participants in SABRE aged between 58 and 85 years in the second wave of follow-up with the aim of providing information on the feasibility and reproducibility of detailed measures of cardiovascular structure and function in a community-based study of older people.

## Methods

### SABRE Cohort

The design of SABRE has been described in detail previously (2). Briefly, all traceable surviving participants from the baseline studies conducted in 1988 to 1991 were invited to attend the second wave of follow-up at St Mary's Hospital, London. Of 3,410 survivors, 1,438 (42%) participants attended the clinic between 2008 and 2011 (69.6 ± 6 years). Participants attended for clinical investigations as listed in Supplementary Table 1. They also completed a health and lifestyle questionnaire and were asked for consent to primary care medical record review. All participants gave written informed consent and the study was approved by the local research ethics committee.

### Echocardiographic Measurements

The SABRE study employed standard and advanced echo techniques that characterized (1) left ventricular (LV) structure and geometry, (2) LV systolic function, (3) LV dyssynchrony, (4) LV diastolic function, and (5) left atrial (LA) structure. The incremental value of LV mechanics by 3D-speckle-tracking echocardiography (3D-STE) in predicting adverse outcomes is of particular interest, considering that SABRE is the first multi-ethnic longitudinal study to capture 3D-STE measures.

#### Data Acquisition Protocol

All participants underwent a transthoracic echocardiographic examination using a Philips iE33 equipped with an S5-1 phased array ultrasound transducer for 2D and Doppler imaging and a matrix array (X3-1) transducer for 3D data acquisition. A brief outline of the SABRE echocardiography imaging protocol is listed in Table 1. The examination was performed by two experienced sonographers in accordance with the ASE guidelines (4). Echocardiographic measures were obtained from the parasternal long-axis (PLAX); parasternal short-axis; and apical four-, two-, and five-chamber views. Optimal images were attained prior to recording and harmonic imaging was used. An adequate quality of ECG signal was ensured throughout the examination. For 2D-images and spectral-Doppler imaging, 3–5 and 10 cardiac cycles were recorded, respectively.

**Table 1:** SABRE echocardiographic imaging protocol.

**Parasternal position**	
Parasternal long axis	- 2D imaging of LV ▪ 3 cardiac cycles - 2D imaging, zoomed on LVOT - M-mode imaging focused on LA ▪ 10 cardiac cycles with a sweep speed of 50 ms - Color Doppler of mitral and aortic valves
Parasternal short axis - Mitral valve level - Papillary muscle level - LV apex	- 2D imaging ▪ 5 cardiac cycles
**Apical position**	
Apical four-chamber view	- 2D imaging - 2D imaging, focused/zoomed on LV ▪ 5 cardiac cycles - PW Doppler of mitral inflow ▪ 2–5 mm SV ▪ 50–100 mm/s sweep speed ▪ 10 cardiac cycles - Tissue Doppler imaging of septal and lateral mitral valve annulus - 3D full volume of acquisition of LV ▪ Four sub-volumes over 4 cardiac cycles
Apical five-chamber view	- 2D imaging - Color Doppler of aortic valve - CW Doppler of trans-aortic flow ▪ 10 cardiac cycles
Apical two-chamber view	- 2D imaging focused/zoomed on LV ▪ 5 cardiac cycles - Tissue Doppler imaging of inferior wall

*CW, continuous wave; LA, left atrium; LV, left ventricle; LVOT, LV outflow tract; PW, pulse wave*.

Depth, sector width, and gain setting were included in 2D image optimization. Harmonic imaging was used to ensure clear visualization of endocardial borders for quantitative analysis including volumes and 2D-STE analysis. Parallel ultrasound beam orientation to the blood flow of interest was ensured for all Doppler acquisitions with a sample volume size 2–5 mm for PW Doppler and a sweep speed between 50 and 100 mm/s.

For 3D-imaging, a LV full-volume dataset was obtained from the apical position. Depth and sector width setting and gain were adjusted as needed (5). Four sub-volumes acquired over four cardiac cycles during held respiration were obtained in the wide-angled (93° × 80°) acquisition mode. The presence of an acceptable ECG signal was checked before accepting the loop. Scans were stored on a secure fileserver in DICOM or native format.

#### Data Analysis

All conventional echocardiographic analyses were performed on the ultrasound machine during the clinic visit using Philips QLAB software 7.0, averaging three measurements. A 2D-STE analysis was performed offline using Philips QLAB software 8.1 Tissue Motion Quantification Advanced (TMQA). A 3D-echocardiography (3DE) analysis was performed using Philips QLAB software 7.0 with 3DQ (Cardiac 3D Quantification) for LV mass and 3DQ Advanced for LV volumes. A 3D-STE LV myocardial deformation analysis was performed using TomTec 4D LV-Analysis (TomTec Imaging Systems, Munich, Germany). These were done shortly following the completion of the study except for 3D-STE, which was performed more recently.

Measured echocardiographic parameters are listed in Table 2. LV and LA dimensions, outflow tract diameter, and LV wall thickness from 2D-guided M-mode were measured from the PLAX from which LV mass was calculated, following the ASE recommendations (6). LV hypertrophy (LVH) based on conventional echocardiography-derived LV mass was defined as LV mass indexed to body surface area (BSA) (7) >115 g/m^2^ in men and 95 g/m^2^ in women (6). Relative wall thickness (RWT) was calculated (6). LV geometry was categorized as follows: normal if RWT ≤ 0.42 and no LVH; LV remodeling if RWT > 0.42 and no LVH; LV concentric hypertrophy if RWT > 0.42 and LVH; and LV eccentric hypertrophy if RWT ≤ 0.42 and LVH (6). LV volumes from conventional echocardiography were calculated by the Teichholz formula using the linear dimensions from which LV ejection fraction (LVEF) was derived to maintain the compatibility with previous sweeps and permit comparisons with other cohort studies while recognizing it is not the best method (6). Midwall fractional shortening was also calculated (8, 9). Tissue Doppler analysis of lateral and septal mitral annulus motion was performed, and peak longitudinal systolic velocity (*s*′) and peak early and late mitral annular relaxation velocities (*e*′ and *a*′) were calculated. Mitral inflow early(E wave) and late (A wave) diastolic velocities were measured by PW Doppler with a sample volume placed at the tip of mitral valve leaflets (10). *E*/*e*′ was calculated as an index of LV filling pressure (10).

**Table 2:** SABRE echocardiographic measures.

	**Primary measures**	**Derived measures**
LV structure	LV wall thickness, IVSd, and PWTd (PLAX) LV internal-diastolic dimension (PLAX) LV internal-systolic dimension (PLAX) LV end-diastolic volume (3D) LV end-systolic volume (3D) LV SV (3D) LV CO (3D) LV mass (3D)	LVEDV^*^ (ml) = (0.7/2.4 + LVIDd) × LVIDd LVESV^*^ (ml) = (0.7/2.4 + LVIDs) × LVIDs SV (ml) = LVEDV – LVESV and indexed to BSA SV index (ml/m^2^) = SV/BSA CO (ml/min) = SV × HR CO index (ml/min/m^2^) = SV × HR LV mass (g) = 0.8 × {1.04 [(LVIDd + PWTd + IVSd)^3^ – LVIDd^3^]} LV RWT = 2 × PWd/LVIDd
LV systolic function	Septal and lateral TDI *S*′ (A4C) Inferior TDI *S*′ (A2C) LV EF (3D) GLS (3D) GCS (3D) Twist and rotations (3D) Average segmental longitudinal, circumferential, radial, and principle tangential strain (3D)	Endocardial fractional shortening (%) = 100 × (LVIDd – LVIDs)/LVIDd Mid-wall fractional shortening = $\frac{(\text{lvidd}+\frac{\text{ivsd}}{2}+\frac{\text{pwtd}}{2})-\sqrt[{3}]{{\left(\text{lvidd}+\frac{\text{ivsd}}{2}+\frac{\text{pwtd}}{2}\right)}^{3\;}-\text{ lvidd}+{\text{lvids}}^{3}}}{\text{lvidd}+\frac{\text{ivsd}}{2}+\frac{\text{pwtd}}{2}}$ LV EF (%) = 100 × (LVEDV – LVESV)/LVEDV
LV diastolic function	E wave, A wave, and deceleration time (A4C) Septal and lateral TDI *e*′ and *a*′ (A4C) Inferior TDI *e*′ and *a*′ (A2C)	*E*/*A* ratio = E wave/A wave *e*′/*a*′= TDI *e*′/TDI *a*′ *E*/*e*′ ratio = E wave/TDI *e*′ Peak untwisting rate, °/s
LV dyssynchrony		SDI _volume−based_ (3D) (defined as the standard deviation of time to minimum segmental volumes over 16 LV segments) Strain-based systolic dyssynchrony indices (calculated as the standard deviation of time to peak segmental strain over 16 LV segments) volume- and strain-derived dispersion indices
LV-arterial interaction		Total peripheral resistance = mean arterial/CO Total peripheral compliance = SV/pulse pressure Pressure volume ratio = end-systolic pressure/LVESV Effective arterial elastance = end-systolic pressure/SV
LA structure & function	LA A-P dimension (PLAX)	
Valvular function	Aortic Valve: AV peak velocity, and VTI (A5C)	

* *Calculated by the Teichholz formula. A4C, apical four-chamber view; A2C, apical two-chamber view; A5C, apical 5-chamber; A-P, anteroposterior; AV, aortic valve; BSA, body surface area; CO, cardiac output; e′, peak early diastolic mitral annular velocity; EF, ejection fraction; GCS, global circumferential strain; GLS, global longitudinal strain; HR, heart rate; IVSd, interventricular septal diastolic wall thickness; LA, left atrial; LV, left ventricular; LVEDV, LV end-diastolic volume; LVESV, LV end-systolic volume; LVIDd, LV internal-diastolic dimension; LVIDs, LV internal-systolic dimension; PWTd, posterior diastolic wall thickness; PLAX, parasternal long-axis view; s′, peak systolic mitral annular velocity; RWT, relative wall thickness; SDI, systolic dyssynchrony index; SV, stroke volume; TDI, tissue Doppler imaging; VTI, velocity-time integral*.

3DE LV mass and volumetric analyses were performed offline in accordance with a pre-developed protocol. Only 3D datasets of the highest quality free of stitching artifacts were used. All views were optimized to ensure that the LV was not foreshortened. For LV mass, the endocardial and epicardial boundaries of the apical four- and two-chamber views were traced at end-diastole (ED), the first frame after mitral valve closure, and end-systole (ES), the first frame after aortic valve closure. Cardiac 3DQ calculated LV mass using the biplane method of discs. LVH based on 3DE-derived LV mass was determined using the 95th upper percentiles of indexed LV mass reported by the Multi-Ethnic Study of Atherosclerosis study (11). Cardiac 3DQ Advanced was used to measure 3D LV volumes without the geometrical assumptions of 2D methods. Five reference points were selected on the LV in two orthogonal views in each ED and ES frames. The software then automatically tracked the endocardium from frame to frame throughout the cardiac cycle to calculate the volumes and LVEF and constructed a 3D shell of the LV and global and regional volumetric waveforms. Manual adjustments were performed when needed.

LV advanced myocardial mechanics were assessed according to a pre-specified protocol. Acceptable image quality was defined as follows:

- **Good(score-1)**=clear visualization of endocardium in all 16 segments
- **Fair(score-2)**=unclear visualization of endocardium in ≤2 segments or presence of minor artefacts e.g. apical noise.
- **Adequate(score-3)**=unclear visualization of endocardium in ≤6 segments.
- **Poor(score-4)**=unclear visualization of endocardium in >6 segments, but reliable tracking throughout the cardiac-cycle using the adjacent segments as a reference.

Unacceptable image quality was defined as follows:

1. Major stitching artifacts preventing reliable tracking of the endocardium.
2. Unacceptable visualization of the endocardial boundaries.
3. Multiple segments (≥4 = ≥25%) of the LV wall being outside of the image sector.

The identity of non-visible segments were recorded in a spreadsheet during analysis as some regions are known to be less feasible, such as the apex and anterior and anterolateral segments (12). A 16-segment model was used and LV segments were scored as 0 if non-visible and 1 if visible (6).

LV endocardial borders at ED were automatically tracked and manual adjustments were performed when needed (12, 13). LV volumes, including LVEF and systolic dyssynchrony index (SDI), global longitudinal strain (GLS), and global circumferential strain (GCS); average peak segmental longitudinal, circumferential, radial, and principle tangential strains; and twist and rotational indices were calculated by the software. Additional measures were derived: (1) strain-based systolic dyssynchrony indices, calculated as the standard deviation of time to peak segmental strain over 16 LV segments normalized to cardiac cycle length, and (2) volume- and strain-derived dispersion indices (Di), defined as difference between the minimum and maximum time to peak of a measure over 16 LV segments normalized to cardiac cycle length (14).

### Vascular Measurements

#### Pulse Wave Velocity

Pulse wave velocity (PWV) is a gold-standard measure of arterial stiffness (15). The assessment of PWV relies on the determination of pulse transit time (TT), defined as the time the pulse wave takes to travel from one point to another over an arterial segment of length (*L*).


$$\begin{array}{l} \text{PWV}=L/\text{TT }\left(\text{m}/\text{s}\right) \end{array}$$

The distance from the sternal notch to the common femoral artery (mm) at the inguinal ligament was measured using a measuring tape while the participants lay in a supine position.

A PT2000 (Micro Medical Ltd, Kent) was used to acquire 10 sequential blood velocity waveforms at the carotid and femoral arteries using a 4-MhZ CW directional Doppler probe with a zero crossing detector and the ECG R wave as a time reference. At least two windows of good quality flow traces were recorded.

#### Central Blood Pressure and Pulse Wave Analysis

Aortic (central) blood pressure (BP) differs from brachial (peripheral) BP and may be a superior predictor of cardiovascular events (16). Radial artery applanation tonometry was assessed using the SphygmoCor device (ATCOR, Sydney, Australia) to record at least six cardiac cycles of central BP waveforms, estimated after calibrating the device to resting brachial systolic and diastolic BP (17).

#### Coronary Artery Calcification

Computed tomography (CT) coronary artery calcium scores (CACS) were measured in those without stents using a Philips Mx8000 IDT 64 detector scanner (2.5-mm slice thickness and 25-mm increments). Images were transferred to a workstation for offline analysis (HeartBeat CS) by an experienced radiographer blinded to participant identity. Areas of calcification were assigned to the four main territories of the coronary arteries: left main, left anterior descending, circumflex, and right coronary artery.

#### Common Carotid Intimal Medial Thickness

Vascular ultrasound of the left carotid artery was performed using Philips iE33 equipped with a linear array transducer (L11-3). Defult settings (FR = 50, depth = 2.5 cm, gain = 62 dB, *C* = 51 dB, and *P* = low and pen) were used, but adjustments were allowed to optimize image quality. A cine loop of at least five cycles at three angles (lateral, posterior, and anterior) as well as one still image captured at the R wave for each angle were acquired. Data were analyzed offline using the AMS-II (v1.1364) (18). The region of interest was defined as 1 cm proximal to the carotid bulb with the analyzed image chosen to ensure optimal quality of the far wall. Modifications of the detected borders or length were performed when necessary. Carotid plaque was defined as the presence of focal wall thickening at least 50% greater than that of the surrounding vessel wall or as a focal region with carotid intimal medial thickness (cIMT) <1.5 mm protruding into the lumen distinct from the adjacent boundary (19). Plaque characteristics including area, gray-scale median, and percentage white were measured using the AMS-II software.

#### Retinal Imaging

Fundus imaging of both eyes was performed in people without glaucoma or any condition that prevented adequate retinal imaging using a Zeiss FF450+ fundus camera and an Oscar 510C-CCD after the administration of eye drops (1% tropicamide and 2.5% phenylephrine). Refraction was measured using a Nidek AR-310 auto-refractometer. Retinopathy was defined according to the NHS Diabetic Eye Screening Programme, and quantitative measures of the retinal microvasculature were performed as described (20). Feasibility and reproducibility have been reported previously (20).

### Quality Control and Reproducibility Analyses

All clinic staff underwent training and accreditation, and data quality was monitored regularly throughout the study. To quantify the repeatability and reproducibility of echo measurements, two sonographers independently analyzed the same echocardiograms in subsets of randomly selected participants (*n* = 10 for conventional echocardiographic measures and *n* = 20 for 3DE measures) with an interval of at least 2 weeks to avoid recall bias and blinded to the first observer measurements. Test-retest reproducibility of conventional and 3D-STE analysis was performed in selected participants who agreed to re-attend the clinic 4–8 weeks after the first visit (adequate images for analysis were available in *n* = 36 for conventional echocardiographic measures and *n* = 10 for 3DE measures). The repeatability and reproducibility of vascular measurements was performed following the same approach in at least 10 randomly selected subjects.

#### Statistical Analysis

Repeated measurements were assessed by intraclass correlation coefficient (ICC) calculated by linear mixed modeling. Reliability was classified as follows: ICC < 0.4 = poor, 0.4 ≥ ICC < 0.75 = fair to good, and ICC ≥ 0.75 = excellent (21). Agreement was measured by Bland–Altman (BA) analysis and reported as the mean difference with 95% limits of agreement. All BA graphs of the intra-observer (repeatability) and inter-observer (reproducibility) reliability of the echocardiographic and vascular measures are shown in the online Supplementary Material. All analyses were performed in Stata 15.1 (StataCorp LLC, United States).

## Results

### Feasibility

Brief characteristics of the population are summarized in Table 3.

**Table 3:** Characteristics of SABRE participants (*n* = 1,438; visit 2).

**Characteristics**	
Age (years), mean ± SD	69.7 ± 6.2
Male, *n* (%)	1,092 (75.9)
Europeans/South Asians/African Caribbeans, *n* (%)	684 (47.6)/522 (36.3)/232 (16.1)
Height (cm), mean ± SD	168.0 ± 8.6
Weight (kg), mean ± SD	78.0 ± 15.0
Body mass index (kg/m^2^), mean ± SD	27.6 ± 4.8
Hypertension, *n* (%)	965 (67.1)
Diabetes, *n* (%)	451 (31.4)
Coronary heart disease, *n* (%)	362 (25.2)
Stroke, *n* (%)	81 (5.6)
Disability, *n* (%)	569 (39.8)
Smoking status (never/ex/current), *n* (%)	810 (56.7)/529 (37.0)/90 (6.3)

*Disability was defined as a get up and go test time ≥12 s*.

#### Echocardiography

**Figure 1:**
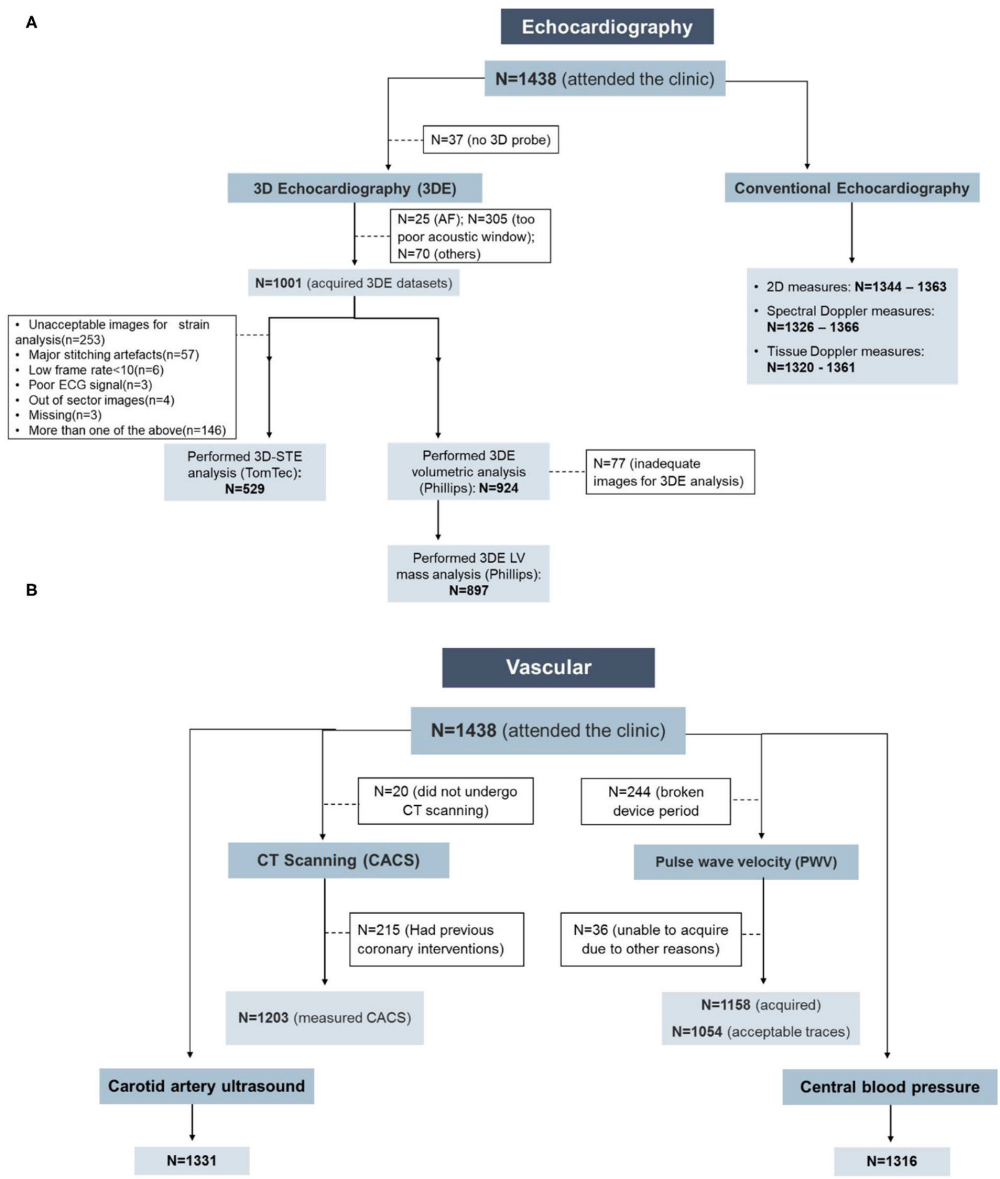
Feasibility of the cardiovascular measurements in the Southall and Brent Revisited (SABRE) study. **(A)** Echocardiography and **(B)** vascular measurements. AF, atrial fibrillation; CACS, coronary artery calcium score; CT, computed tomography.

In up to 95% of clinic attendees, 2D, spectral- and tissue-Doppler echocardiography was analyzable (Table 4, Figure 1A). In participants with atrial fibrillation or poor image quality, 3DE was not acquired, and there was a small subset of participants who had attended the clinic before the 3D probe became available; these were excluded from the denominator when feasibility was estimated. 3DE was acquired in 71% of all clinic attendees and, using QLAB, 924 (92%) had successful volumetric analysis and 897 (89.6%) had LV mass calculated. The difference in these numbers reflects difficulties in tracking the epicardium compared to the endocardium. Fifty three percent of those who had 3DE datasets had 3D deformation measurements by TomTec. Reasons for non-analysis were unacceptable images for strain analysis (*n* = 253, 53.6%), out-of-sector images (*n* = 4, 0.85%), stitching artifacts (*n* = 57, 12.1%), low frame rate (*n* = 6, 1.3%), poor ECG signal (*n* = 3, 0.64%), missing (*n* = 3, 0.64%), or composite (*n* = 146, 30.9%). The feasibility of 3D-STE was 38% of all clinic attendees.

**Table 4:** Feasibility of the cardiovascular measures in 1,438 SABRE participants.

**2D**	
LVIDd	1,354 (94%)
LVIDs	1,352 (94%)
IVSd	1,354 (94%)
IVSs	1,352 (94%)
PWd	1,354 (94%)
PWs	1,353 (94%)
LA diameter	1,344 (93%)
LVOT diameter	1,363 (95%)
**Spectral-Doppler**	
AV VTI	1,355 (94%)
AV max velocity	1,358 (94%)
E wave	1,366 (95%)
A wave	1,326 (92%)
Deceleration time	1,360 (95%)
**Tissue-Doppler**	
*e'* septal	1,359 (95%)
*a'* septal	1,320 (92%)
*s'* septal	1,362 (95%)
*e'* lateral	1,360 (95%)
*a'* lateral	1,321 (92%)
*s'* lateral	1,361 (95%)
*E/e'*	1,337 (93%)
**3DE**	
QLAB EF, EDV, ESV	924 (92%)
QLAB LV mass	897 (89.6%)
3D-STE^*^	
GLS, GCS	529 (53%)
Twist and rotations	529 (53%)
Vascular^*^	
cIMT	1,331 (92.5%)
Central SBP and DBP	1,316 (91.5%)
AIx,	1,316 (91.5%)
Total CACS	1,203 (83.7%)
PWV	1,054 (91%)

* *See text in the manuscript for details.*

*AV, aortic valve; AIx, augmentation index; CACS, coronary artery calcification score; cIMT, common carotid intimal medial thickness, DBP, diastolic blood pressure; EDV, end-diastolic volume; ESV, end-systolic volume; EF, ejection fraction; GCS, global circumferential strain; GLS, global longitudinal strain; IVSd, diastolic interventricular septal thickness; IVSs, systolic interventricular septal thickness; LA, left atrial; LV, left ventricle; LVIDd, diastolic left ventricular internal diameter; LVIDs, systolic left ventricular internal diameter; LVOT, left ventricular outflow tract; PWTd, diastolic posterior wall thickness; PWTs, systolic posterior wall thickness; PWV, pulse wave velocity; SBP, systolic blood pressure; VTI, velocity time integral*.

**Figure 2:**
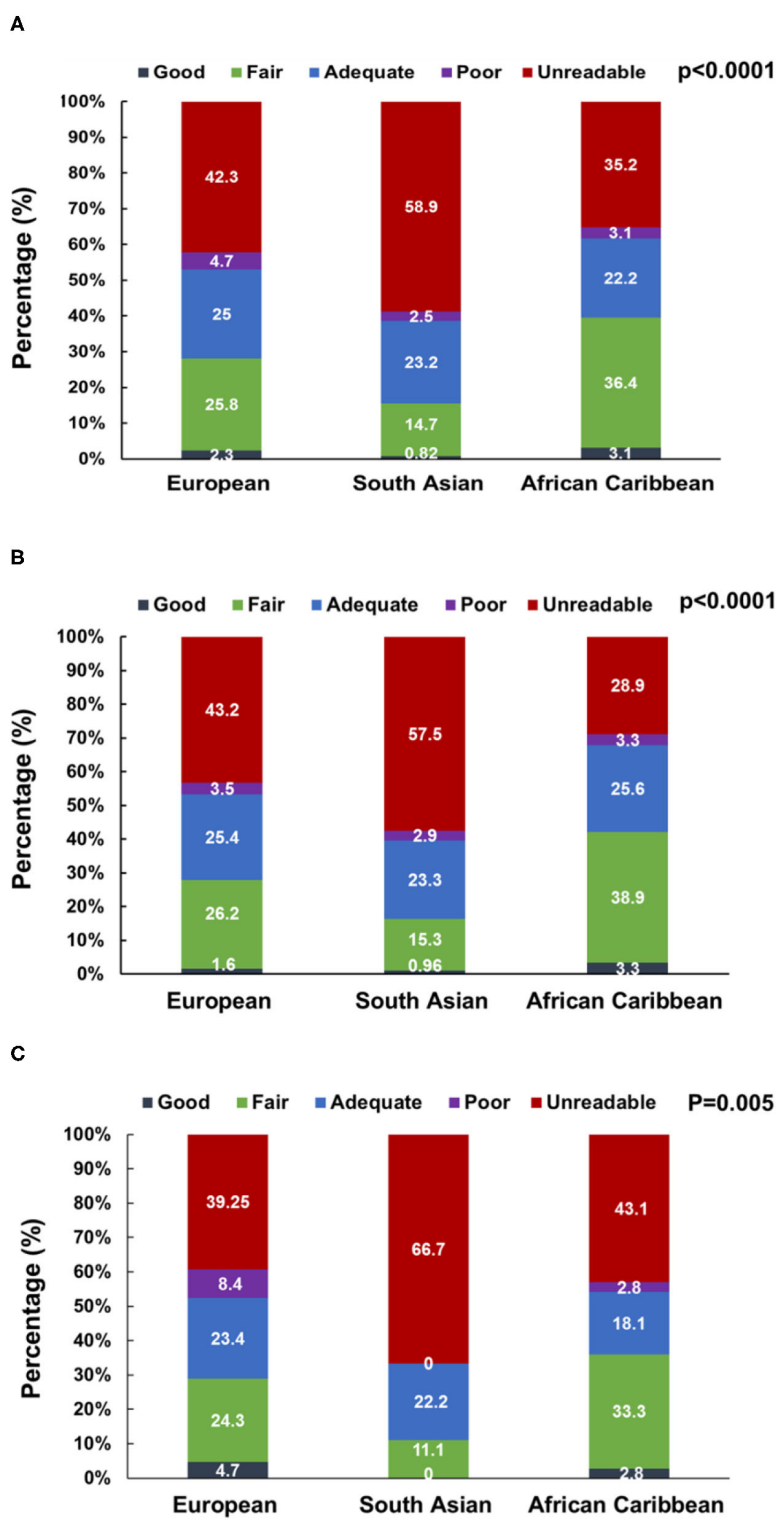
3D dataset image quality score stratified by ethnicity in the overall SABRE population [*N* = 1,001, **(A)**] and among men [*N* = 768, **(B)**] and women [*N* = 233, **(C)**] participants. Numbers are percentages.

The quality of the 3D datasets was scored as good, fair, adequate, poor, and unacceptable in 1.9, 23.5, 23.9, 3.6, and 47.2% of participants, respectively. When stratified by ethnicity (Europeans, South Asians, and African Caribbean), 59% (*n* = 216) of South Asian participants had unacceptable 3D images with fewer participants having good (1%, *n* = 3) and fair (15%, *n* = 54) quality 3D images. In contrast, only 35% (*n* = 57) African Caribbean participants had unacceptable 3D images with more participants having good (3%, *n* = 5) and fair (36%, *n* = 59) quality 3D images (*P* < 0.0001, *n* = 1,002, Figure 2). Broadly similar trends were observed in men (*P* < 0.0001, *n* = 768) and women (*P* = 0.005, *n* = 233); however, in South Asians, there were more women with unreadable 3D images compared to men (67 vs. 58%, Figure 2).

#### Vascular Measurements

Central BP, cIMT, and CACS showed high feasibility with good quality measurements achievable in 92, 93, and 84% of clinic attendees, respectively (Figure 1B; Table 4). PWV was not collected in 244 participants due to device failure, and excluding this reason for missing data gave a feasibility of 97%. CACS was measurable in all participants who underwent CT scanning; 15% were ineligible due to stent placement, and 1% of eligible individuals did not undergo CT scanning either due to refusal or unavailability of the scanner.

### Repeatability and Reproducibility

#### Echocardiography

Intra-observer (repeatability) and inter-observer (reproducibility) reliability of conventional echocardiographic measures was good or excellent for both read-reread and test-retest studies (Table 5).

**Table 5:** Reproducibility of conventional echocardiographic measures.

	**Bland–Altman Analysis [Mean_Diff_ (95% LOA), ICC]**					
	**Intra-observer (*****n*** **=** **10)**		**Inter-observer (*****n*** **=** **10)**		**Test-retest (*****n*** **=** **36)**	
**2D measures**						
LA dimension, cm	−0.02 (−0.17, 0.13)	0.97	−0.06 (−0.25, 0.12)	0.98	−0.001 (−0.23, 0.23)	0.98
IVSd, cm	−0.01 (−0.11, 0.08)	0.91	−0.07 (−0.33, 0.17)	0.85	−0.01 (−0.16, 0.14)	0.94
LVIDd, cm	0.001 (−0.23, 0.23)	0.96	−0.007 (−0.20, 0.18)	0.97	0.001 (−0.34, 0.34)	0.95
PWTd, cm	−0.01 (−0.13, 0.10)	0.96	−0.03 (−0.16, 0.09)	0.82	−0.02 (−0.25, 0.22)	0.70
IVSs, cm	−0.007 (−0.09, 0.08)	0.92	0.005 (−0.09, 0.10)	0.96	0.003 (−0.22, 0.23)	0.92
LVIDs, cm	0.04 (−0.17, 0.26)	0.97	0.02 (−0.16, 0.21)	0.96	0.01 (−0.43, 0.46)	0.93
PWTs, cm	−0.04 (−0.21, 0.12)	0.93	0.01 (−0.30, 0.33)	0.77	−0.001 (−0.21, 0.21)	0.88
LVOT diameter, cm	−0.02 (−0.14, 0.10)	0.96	−0.007 (−0.10, 0.09)	0.96	0.01 (−0.2, 0.21)	0.86
**Spectral-Doppler measures**						
AV peak velocity, m/s	−3.8 (−9.9, 2.3)	0.97	0.001 (−0.08, 0.08)	0.99	0.02 (−0.25, 0.28)	0.88
AV VTI, cm	0.14 (−2.8, 3.1)	0.96	0.32 (−6.1, 6.8)	0.87	0.11 (−7.0, 7.3)	0.86
E wave, cm/s	1.5 (−4.1, 7.2)	0.98	−2.3 (−6.7, 2.1)	0.95	0.35 (−17.6, 18.3)	0.84
A wave, cm/s	−0.53 (−11.2, 10.2)	0.91	−1.4 (−4.7, 1.7)	0.99	0.23 (−14.7, 15.2)	0.90
Deceleration time, ms	10.9 (−34.2, 56.0)	0.82	7.4 (−18.2, 33.0)	0.97	−2.9 (−87.1, 81.3)	0.58
**Tissue-doppler measures**						
*s*′, cm/s	−0.30 (−1.4, 0.81)	0.95	0.24 (−0.62, 1.1)	0.95	−0.20 (−1.65, 1.25)	0.89
*e*′, cm/s	0.04 (−0.9, 1.0)	0.91	−0.30 (−1.2, 0.58)	0.98	−0.43 (−3.5, 2.6)	0.66
*a*′, cm/s	0.16 (−0.79, 1.1)	0.98	−0.24(−0.81, 0.33)	0.99	−0.24 (−3.1, 2.7)	0.78

*Data are mean difference [95% limit of agreement (LOA)]. Abbreviations as in Table 4 in addition to: ICC, interclass correlation coefficient*.

The 3D volumetric analysis using QLAB software showed excellent reliability for all measures. Mean difference [95% limit of agreement (LOA)] and ICC of the inter-observer difference were −0.06 (−5.5, 5.4) and 0.82 ICC for LVEF (%); −0.1 (−8.2, 8.0) and 0.96 ICC for end-diastolic volume (ml); 0.0 (−4.6, 4.6) and 0.96 ICC for end-systolic volume (ml); and 1.3 (−7.5, 10.2) and 0.98 ICC for LV mass (g). The intra-observer difference for LV mass was −3.5 (−18.2, 11.1) and 0.96 ICC (g).

LV volumes, LVEF, and LV mass derived from 3D-STE analysis using TomTec showed excellent repeatability and reproducibility (Table 6). Global and averaged peak segmental LV strain measures also showed excellent repeatability and reproducibility, except for longitudinal strain measures, which showed fair to good inter-observer variability. Rotational measures showed fair to good repeatability and reproducibility. All SDIs showed good to excellent repeatability and reproducibility. Dispersion indices also were reproducible apart from circumferential strain-Di, which showed poor repeatability and reproducibility. Overall, reproducibility was better for SDIs than for dispersion indices (Table 6).

**Table 6:** Reproducibility of 3D-speckle-tracking echocardiographic analysis.

	**Bland–Altman Analysis [Mean_Diff_ (95% LOA), ICC]**					
	**Intra-observer (*****n*** **=** **20)**		**Inter-observer (*****n*** **=** **20)**		**Test-retest (*****n*** **=** **10)**	
**Volumetric measures**						
EDV, ml	−4.6 (−14.3, 5.0)	0.97	−1.7 (−11.6, 8.2)	0.96	0.88 (−14.4, 16.2)	0.85
ESV, ml	−1.1 (−7.1, 4.7)	0.97	−1.7 (−7.5, 4.0)	0.97	0.23 (−6.6, 7.0)	0.85
EF, %	−0.94 (−4.2, 2.3)	0.96	0.79 (−2.5, 4.1)	0.96	0.34 (−2.5, 3.1)	0.92
SV, ml	−3.4 (−9.6, 2.7)	0.96	−0.03 (−6.7, 6.8)	0.94	0.65 (−8.8, 10.1)	0.87
LV mass, g	2.1 (−8.7, 13.0)	0.97	1.4 (−15.7, 18.6)	0.94	0.42 (−10.5, 11.3)	0.96
**Deformation measures**						
GCS, %	1.19 (−1.0, 3.4)	0.96	0.06 (−2.9, 3.0)	0.93	0.63 (−3.5, 4.7)	0.79
GLS, %	0.48 (−1.8, 2.7)	0.91	−1.3 (−6.2, 3.4)	0.60	−0.84 (−4.8, 3.2)	0.52
Peak CS, %	1.03 (−1.5, 3.6)	0.94	0.13 (−3.5, 3.8)	0.89	0.67 (−4.7, 6.0)	0.64
Peak LS, %	0.43 (−2.07, 2.9)	0.88	−1.5 (−6.1, 2.9)	0.60	−1.0 (−5.6, 3.6)	0.44
Peak PT, %	1.11 (−1.6, 3.8)	0.93	−0.29 (−4.4, 3.8)	0.84	0.2 (−3.1, 3.5)	0.77
Peak RS, %	−1.22 (−4.2, 1.7)	0.95	1.3 (−4.3, 6.9)	0.82	0.54 (−2.1, 3.1)	0.87
**Rotational measures**						
Peak basal rotation, °	0.10 (−3.3, 3.5)	0.86	−0.65 (−5.3, 4.0)	0.71	0.06 (−7.7, 7.8)	0.35
Peak apical rotation, °	0.33 (−5.3, 6.0)	0.64	1.0 (−4.1, 6.1)	0.69	0.65 (−4.1, 5.4)	0.81
Peak twist, °	0.27 (−8.0, 8.6)	0.73	1.7 (−6.9, 10.4)	0.69	0.66 (−9.8, 11.2)	0.74
Torsion, °/cm	0.04 (−0.97, 1.07)	0.73	0.22 (−0.88, 1.3)	0.67	0.08 (−1.2, 1.4)	0.69
**Systolic dyssynchrony indices**						
SDI _volume−based_, %	0.10 (−0.30, 0.50)	0.98	−0.02 (−0.79, 0.75)	0.96	−0.22(−3.2, 2.8)	0.61
CS SDI, %	0.11 (−1.9, 2.1)	0.65	0.003 (−1.9, 1.9)	0.63	−0.79 (−3.2, 1.6)	0.72
LS SD, %	0.08 (−1.5, 1.7)	0.82	−0.30 (−1.6, 1.02)	0.89	−0.5 (−3.6, 2.5)	0.82
PTS SDI, %	0.17 (−0.87, 1.2)	0.93	−0.14 (−1.7, 1.4)	0.83	−0.2 (−2.8, 2.4)	0.79
RS SDI, %	0.22 (−1.06, 1.5)	0.85	0.08 (−1.09, 1.26)	0.87	−0.4 (−2.8, 1.9)	0.81
Di _volumes_, %	0.15 (−1.4, 1.7)	0.95	−0.30 (−2.2, 1.6)	0.94	−0.67 (−10.3, 8.9)	0.29
CS Di, %	0.70 (−10.2, 11.6)	0.15	0.31 (−10.1, 10.7)	0.33	−2.7 (−17.5, 12.0)	0.008
LS Di, %	0.41 (−5.6, 6.4)	0.80	−1.3 (−5.9, 3.3)	0.89	−2.2 (−10.3, 5.9)	0.77
PTS Di, %	−0.32 (−5.0, 4.4)	0.79	−2.2 (−10.8, 6.3)	0.47	0.54 (−7.3, 8.3)	0.73
RS Di, %	0.25 (−4.2, 4.7)	0.70	−0.58 (−5.4, 4.3)	0.73	−0.74 (−8.3, 6.8)	0.69

*Data are mean difference [95% limit of agreement (LOA)]. CS, circumferential strain; Di, dispersion; EDV, end-diastolic volume; ESV, end-systolic volume; EF, ejection fraction; GCS, global circumferential strain; GLS, global longitudinal strain; ICC, intra-class correlation coefficient; LS, longitudinal strain; LV, left ventricular; PTS, principle tangential strain; RS, radial strain; SDI, systolic dyssynchrony index; SV, stroke volume*.

Test-retest reproducibility of 3D-STE-derived measures was overall fair to good, but lower than when re-reading images were used as a measure of reliability (Table 6). Nevertheless, volumetric measures showed excellent reproducibility. All strain and rotational measures showed fair to good or excellent test-retest reproducibility, except for longitudinal strain and basal rotational measures, which showed fair and poor test-retest reproducibility, respectively. SDIs showed good to excellent test-retest reproducibility, which was overall better than dispersion indices.

#### Vascular Measurements

PWV, CACS, and cIMT showed fair to good, excellent, and excellent reproducibility, respectively. Test-retest reproducibility of PWV was done in 21 individuals. Mean difference (95% LOA) and ICC were −0.27 (−3.9, 3.3) (m/s) and 0.71 for PWV and 0.83 (−88.2, 89.9) (mm) and 0.54 for the carotid-femoral length measure. Repeatability and reproducibility of CACS were done in 20 individuals and were excellent. The within-observer difference was −84.5 (−396.6, 565.7) and 0.99 ICC and the between-observer difference was −80.8 (−555.9, 394.3) and 0.99 ICC. Repeatability and reproducibility of cIMT and plaque characteristics (area and gray-scale median values) were done in 10 individuals (for cIMT) and *n* = 17 (for read-reread analysis of plaque characteristics) and were excellent, while percentage white had fair to good repeatability and reproducibility (Table 7).

**Table 7:** Reproducibility of carotid intima medial thickness (cIMT) measures.

	**Mean_**Diff**_ (95% LOA)**	**ICC**	**Mean_**Diff**_ (95% LOA)**	**ICC**
	**Intra-observer**		**Inter-observer**	
cIMT^*^ far wall maximum of means, mm	0.04 (−0.08, 0.17)	0.79	−0.001 (−0.12, 0.12)	0.89
Carotid lumen diameter, mm	0.06 (−0.16, 0.28)	0.98	0.02 (−0.46, 0.49)	0.94
Plaque area^*^, mm^2^	−3.3 (−20.7, 14.1)	0.81	0.3 (−11.5, 12.1)	0.90
Gray-scale median, mm	8.8 (−13.4, 31.0)	0.89	2.5 (−57.2, 47.8)	0.73
Percentage white, %	9.3 (−13.4, 31.9)	0.60	10.9 (−16.6, 38.5)	0.48

*Data are mean difference [95% limit of agreement (LOA)]*.

* *cIMT, n = 10; plaque characteristics, n = 17. ICC, intra-class correlation coefficient*.

## Discussion

Over 1,400 participants attended the SABRE 20-year follow-up and underwent a comprehensive health examination. The feasibility of image acquisition by most conventional echocardiographic measures was high, which is in line with previous reports in other cohort studies (3, 22, 23). By contrast, 3DE had ~71% acquisition feasibility, while 3D-STE feasibility was highly influenced by image quality and only half of the datasets could be analyzed. These proportions are slightly better than those reported by the Atherosclerosis Risk in Communities (ARIC) study (3, 24). The potential bias that may result from this missingness is an important consideration for future studies in older populations using 3D-STE (25–27). We believe this is the first study to show that the feasibility of 3D-STE seems to differ by ethnicity and gender, with the lowest level of feasibility being found in South Asian women and the highest in African Caribbean men. Further studies on this question are needed to establish definitively whether 3D-STE measures are biased by sex and ethnicity relationship to image quality.

Most conventional echocardiographic measurements and 3D-STE-derived LV indices demonstrated good to excellent repeatability/reproducibility, in keeping with other studies (3, 22, 28–32), although previous studies reporting the reproducibility of strain-based dyssynchrony measures by 3D-STE are scarce (14). Test-retest reproducibility, however, tended to be poorer for some measures, a factor that should be considered when performing longitudinal studies (28, 33).

We confirm that vascular measures including cIMT, CACS, and PWV are feasible in large cohort studies (34–38). The analyses of these measures are straightforward and reproducible and have been quite widely used. PWV, however, showed fair to good reliability, in keeping with previous studies (39).

SABRE includes first-generation migrant African Caribbeans and South Asians, who are known to have different risks of CVD compared to White British (40). Capturing advanced and sensitive cardiovascular measures will enable new insights into why this is the case (41–44).

### Limitations and Strengths

SABRE is the largest tri-ethnic UK community-based cohort study and includes a lengthy follow-up period with detailed phenotyping using state-of-the-art methods A large percentage of participants could be traced 20 years later (~90%) and 42% returned for clinic follow-up. However, in common with most longitudinal studies, elderly and/or infirm participants were more likely to decline to attend which may bias our feasibility estimates (Supplementary Table 2). Further, differences in healthcare provision may limit the generalizability of our observations to other countries or settings.

In older participants or those who were obese and diabetic, 3DE was more frequently not feasible, which may also introduce bias (45). This maybe because sicker/frailer patients often have suboptimal echocardiographic image quality, but further studies on this question are needed (33). Improvements in 3D probe technology may improve image quality in future, although some scanning limitations related to morbidities such as atrial fibrillation, body habitus, or inability to lie on an echo couch are likely to remain problematic. While technology and hence quality has improved over time for many of the methods used, the second wave of SABRE employed state-of-the-art approaches for the time when it was conceived and performed. We did not assess any potential effect modification by ethnicity on reproducibility; to be able to assess this, a large sample size would be needed.

## Conclusions

Capturing detailed cardiovascular phenotyping is feasible and reproducible in a large multi-ethnic study of older people. SABRE represents a uniquely rich resource of detailed information on cardiac and vascular structure and function in an ethnically diverse population and should lead to a better understanding of why people of South Asian and African Caribbean ancestry are at elevated risk of cardiometabolic diseases. The information reported in this study will be helpful to other research groups planning similar studies and can inform sample size calculations.

## Data Availability Statement

The datasets presented in this article are not publicly available due to restrictions. Requests for data access should be directed to t.tillin@ucl.ac.uk.

## Ethics Statement

The studies involving human participants were reviewed and approved by St Mary's Hospital Local Research Ethics Committee (07/H0712/109). The patients/participants provided their written informed consent to participate in this study.

## Author Contributions

LA drafted the manuscript and all co-authors critically reviewed and amended the manuscript. All authors contributed to this study by conceiving and designing the study, performing the data collection and the statistical analysis, or assisting in data interpretation.

## Conflict of Interest

The authors declare that the research was conducted in the absence of any commercial or financial relationships that could be construed as a potential conflict of interest.
