# Corrigendum: Imaging Protocol, Feasibility, and Reproducibility of Cardiovascular Phenotyping in a Large Tri-Ethnic Population-Based Study of Older People: The Southall and Brent Revisited (SABRE) Study

**DOI:** 10.3389/fcvm.2021.769050

**Published:** 2021-11-04

**Authors:** Lamia Al Saikhan, Muath Alobaida, Anish Bhuva, Nish Chaturvedi, John Heasman, Alun D. Hughes, Siana Jones, Sophie Eastwood, Charlotte Manisty, Katherine March, Arjun K. Ghosh, Jamil Mayet, Ayodipupo Oguntade, Therese Tillin, Suzanne Williams, Andrew Wright, Chloe Park

**Affiliations:** ^1^Department of Cardiac Technology, College of Applied Medical Sciences, Imam Abdulrahman Bin Faisal University, Dammam, Saudi Arabia; ^2^MRC Unit for Lifelong Health and Ageing, Department of Population Science & Experimental Medicine, UCL Institute of Cardiovascular Science, University College London, London, United Kingdom; ^3^Department of Basic Science, Prince Sultan bin Abdulaziz College for Emergency Medical Services, King Saud University, Riyadh, Saudi Arabia; ^4^National Heart & Lung Institute, Imperial College London and Imperial College Healthcare NHS Trust, Hammersmith Hospital, London, United Kingdom; ^5^Cardio-Oncology Service, Department of Cardiology, Barts Heart Centre, Barts Health NHS Trust, St Bartholomew's Hospital, London, United Kingdom; ^6^Cardio-Oncology Service, Department of Cardiology, University College London Hospital, London, United Kingdom

**Keywords:** population-based, cardiovascular, imaging, echocardiography, vascular, feasibility, reproducibility

In the original article, there was a numerical error in the legend for **Figure 2** as published. The correct legend appears below.

**3D dataset image quality score stratified by ethnicity in the overall SABRE population [*N*** **=** **1,001, (A)] and among men [*N* =** **768, (B)] and women [*N* =** **233, (C)] participants. Numbers are percentages**.

In the original article, there was a **numerical error for some measures** in Table 4 as published. The corrected Table 4 appears below.

**Table 4:** Feasibility of the cardiovascular measures in 1,438 SABRE participants.

**2D**	
LVIDd	1,354 (94%)
LVIDs	1,352 (94%)
IVSd	1,354 (94%)
IVSs	1,352 (94%)
PWd	1,354 (94%)
PWs	1,353 (94%)
LA diameter	1,344 (93%)
LVOT diameter	1,363 (95%)
**Spectral-Doppler**	
AV VTI	1,355 (94%)
AV max velocity	1,358 (94%)
E wave	1,366 (95%)
A wave	1,326 (92%)
Deceleration time	1,360 (95%)
**Tissue-Doppler**	
*e'* septal	1,359 (95%)
*a'* septal	1,320 (92%)
*s'* septal	1,362 (95%)
*e'* lateral	1,360 (95%)
*a'* lateral	1,321 (92%)
*s'* lateral	1,361 (95%)
*E/e'*	1,337 (93%)
**3DE**	
QLAB EF, EDV, ESV	924 (92%)
QLAB LV mass	897 (89.6%)
3D-STE^*^	
GLS, GCS	529 (53%)
Twist and rotations	529 (53%)
Vascular^*^	
cIMT	1,331 (92.5%)
Central SBP and DBP	1,316 (91.5%)
AIx,	1,316 (91.5%)
Total CACS	1,203 (83.7%)
PWV	1,054 (91%)

* *See text in the manuscript for details.*

*AV, aortic valve; AIx, augmentation index; CACS, coronary artery calcification score; cIMT, common carotid intimal medial thickness, DBP, diastolic blood pressure; EDV, end-diastolic volume; ESV, end-systolic volume; EF, ejection fraction; GCS, global circumferential strain; GLS, global longitudinal strain; IVSd, diastolic interventricular septal thickness; IVSs, systolic interventricular septal thickness; LA, left atrial; LV, left ventricle; LVIDd, diastolic left ventricular internal diameter; LVIDs, systolic left ventricular internal diameter; LVOT, left ventricular outflow tract; PWTd, diastolic posterior wall thickness; PWTs, systolic posterior wall thickness; PWV, pulse wave velocity; SBP, systolic blood pressure; VTI, velocity time integral*.

In the original article, there was a numerical error in Figure 1A as published. The corrected Figure 1A appears below.

**Figure 1:**
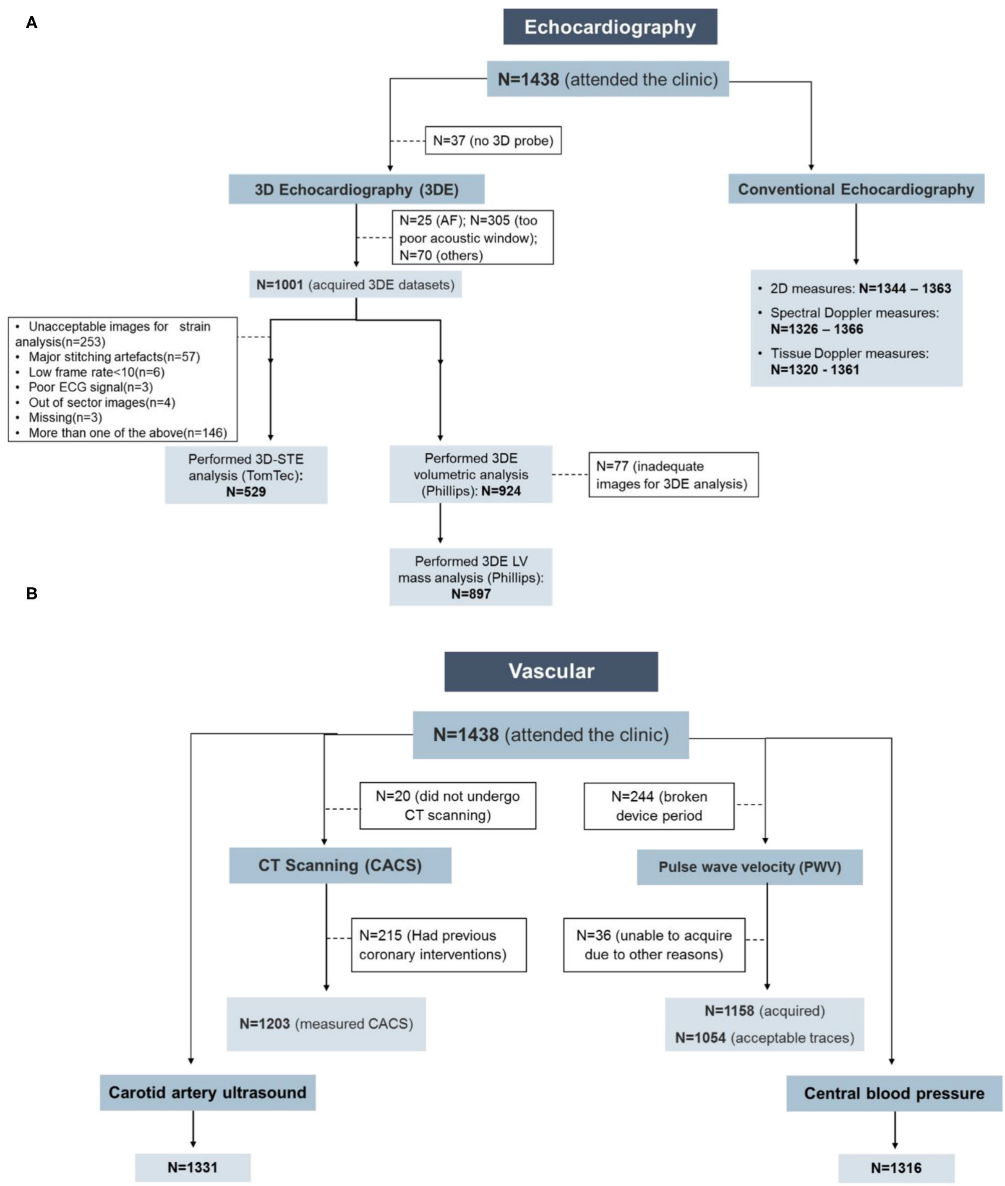


In the original article, there was an error. A correction has been made to **Abstract, Results:**

**Conventional echocardiography and all vascular measurements showed high feasibility (>90% analyzable of clinic attendees), but 3D-echocardiography (3DE) and 3D-STE were less feasible (71% 3DE acquisition feasibility and 38% 3D-STE feasibility of clinic attendees)**.

In the original article, there was an error. A correction has been made to **Results, Echocardiography**, **Paragraph Number 1**:

**3DE was acquired in 71% of all clinic attendees and, using QLAB, 924 (92%) had successful volumetric analysis and 897 (89.6%) had LV mass calculated. The difference in these numbers reflects difficulties in tracking the epicardium compared to the endocardium. Fifty three percent of those who had 3DE datasets had 3D deformation measurements by TomTec**.

In the original article, there was an error. A correction has been made to **Results, Echocardiography**, **Paragraph Number 2**:

**Broadly similar trends were observed in men (*P*** **<** **0.0001**, ***n*** **=** **768) and women (*P* =** **0.005**, ***n*** **=** **233); however, in South Asians, there were more women with unreadable 3D images compared to men (67 vs. 58%, Figure 2)**.

In the original article, there was an error. A correction has been made to **Discussion**, **Paragraph Number 1**:

**By contrast, 3DE had** **~71% acquisition feasibility, while 3D-STE feasibility was highly influenced by image quality and only half of the datasets could be analyzed**.

The authors apologize for this error and state that this does not change the scientific conclusions of the article in any way. The original article has been updated.

## Publisher's Note

All claims expressed in this article are solely those of the authors and do not necessarily represent those of their affiliated organizations, or those of the publisher, the editors and the reviewers. Any product that may be evaluated in this article, or claim that may be made by its manufacturer, is not guaranteed or endorsed by the publisher.

