# Supplementary material for: Imaging Protocol, Feasibility, and Reproducibility of Cardiovascular Phenotyping in a Large Tri-Ethnic Population-Based Study of Older People: The Southall and Brent Revisited (SABRE) Study
Source: Front Cardiovasc Med. 2020 Nov 13;7:591946. doi: 10.3389/fcvm.2020.591946 (PMC7693529; doi:10.3389/fcvm.2020.591946)

Supplementary Material

| **Table S1. List of measurements of SABRE clinic between 2008 and 2011 (*n*=1438)** |
| --- |
| **Anthropometrics** |
| Circumferences: Waist, Hip, Thigh; Height; Leg length |
| **Body fat** |
| Bioimpedance: Percent body fat; Fat mass |
| Computed tomography for fat distribution: Thigh (single slice, mid-thigh); Abdomen (single slice L4) |
| **Bloods** |
| Glucose, insulin, HbA1c |
| Total cholesterol, triglycerides, HDL, cholesterol, LDL |
| Apolipoprotein (metabolomics only) |
| Serum creatinine; Cystatin C |
| Liver function tests: GGT, ALT, ALP |
| Troponin T |
| Adiponectin/leptin |
| Inflammatory markers: CRP, IL-6, NT ProBNP, IL1, IFN, TNF alpha |
| Osteopontin and osteoprotegerin |
| Bilirubin; Globulin; Protein |
| Metabolomics (NMR spectroscopy) |
| Genetics (n= 1200) |
| APOE genotype (n=1210) |
| Stored blood for future analyses: 0.5 ml aliquots, n~=1400; EDTA plasma; Citrated plasma; Serum; Whole blood |
| **2 hour post- glucose challenge (if not known to have diabetes)** |
| Bloods: Glucose, insulin; Metabolomics (NMR spectroscopy) fasting only; Stored post glucose EDTA plasma/serum |
| Urine: Albumin; Creatinine: Stored urines for future analyses |
| **Blood pressure** |
| Resting (oscillometric): Brachial sitting; Brachial lying; Ankle lying; 24 hour ambulatory blood pressure (subset, n=563) |
| Pulsecor (cuff based, sitting) |
| Sphygmocor (applanation tonometry, sitting) |
| **ECG (resting, 12 lead)** |
| **Echocardiography (see details in text)** |
| **Vascular (see details in text)** |
| **Coronary artery calcification (CT)** |
| **Cerebral MRI** |
| White matter lesions, sulcal and ventricular grades; Non-haemorrhagic infarcts and haemorrhagic lesions; Regional brain volumes |
| **Cognitive function testing^1^** |
| Primary variables:  Memory (composite of short-term verbal recall, delayed recall, working memory and visual recognition memory)  Executive function (composite of digits backwards, colour trail B, animal naming)  Secondary variables:  Language (composite of naming, comprehension, repetition)  Orientation in time and space (some CSID items)  Everyday functioning( Medco aspirin sheet)  Community screening in dementia (CSID) (‘global’ score) |
| **Eyes** |
| Retinal photography (4 field, both eyes); Lens photography (opacities and cataracts); Visual acuity |
| **Medical history** |
| Participant recall of major health events(self-completion questionnaire); Primary care medical record review; Hospital Episode statistics;  Self-completion questionnaire: Current socioeconomic circumstances; Health-related quality of life (EQ5D); Activities of daily living; Family history of diabetes/ heart disease; Alcohol; Smoking; Physical activity |

| **Table S2. Baseline (visit 1) characteristics of visit 2 clinic attenders and non-attenders** | | | |
| --- | --- | --- | --- |
|  | **V2 clinic attended** | **V2 clinic not attended** | ***P*-value** |
| Ethnicity, *n*(%) |  |  |  |
| European | 684(48%) | 1662(49%) | 0.6 |
| South Asian | 522(36%) | 1188(35%) |  |
| African Caribbean | 232(16%) | 569(17%) |  |
| Male, *n*(%) | 1092(76%) | 2567(75%) | 0.5 |
| Age, y | 50.0±6.2 | 53.4±6.9 | <0.001 |
| Known diabetes, *n*(%) | 61(4%) | 303(9%) | <0.001 |
| Hypertension, *n*(%) | 126(9%) | 498(15%) | <0.001 |
| Body mass index, kg/m2 | 26.1±3.9 | 26.5±4.1 | <0.001 |
| General Health poor or very poor (self-rated) | 86(6%) | 320(9%) | <0.001 |
| Years of education | 11.7±3.2 | 10.7±3.1 | <0.001 |

Data are means±SD or counts (percentages %).

Known deaths 1989-2008 (i.e. deaths before v2 clinic): 898 (26% of non-clinic attenders).

**Supplementary Figures. B-A graphs for intra- (repeatability) and inter-observer (reproducibility) agreements of the cardiovascular measures**

***Advanced echocardiographic measures***

Strain (Deformation) indices (3D-STE)


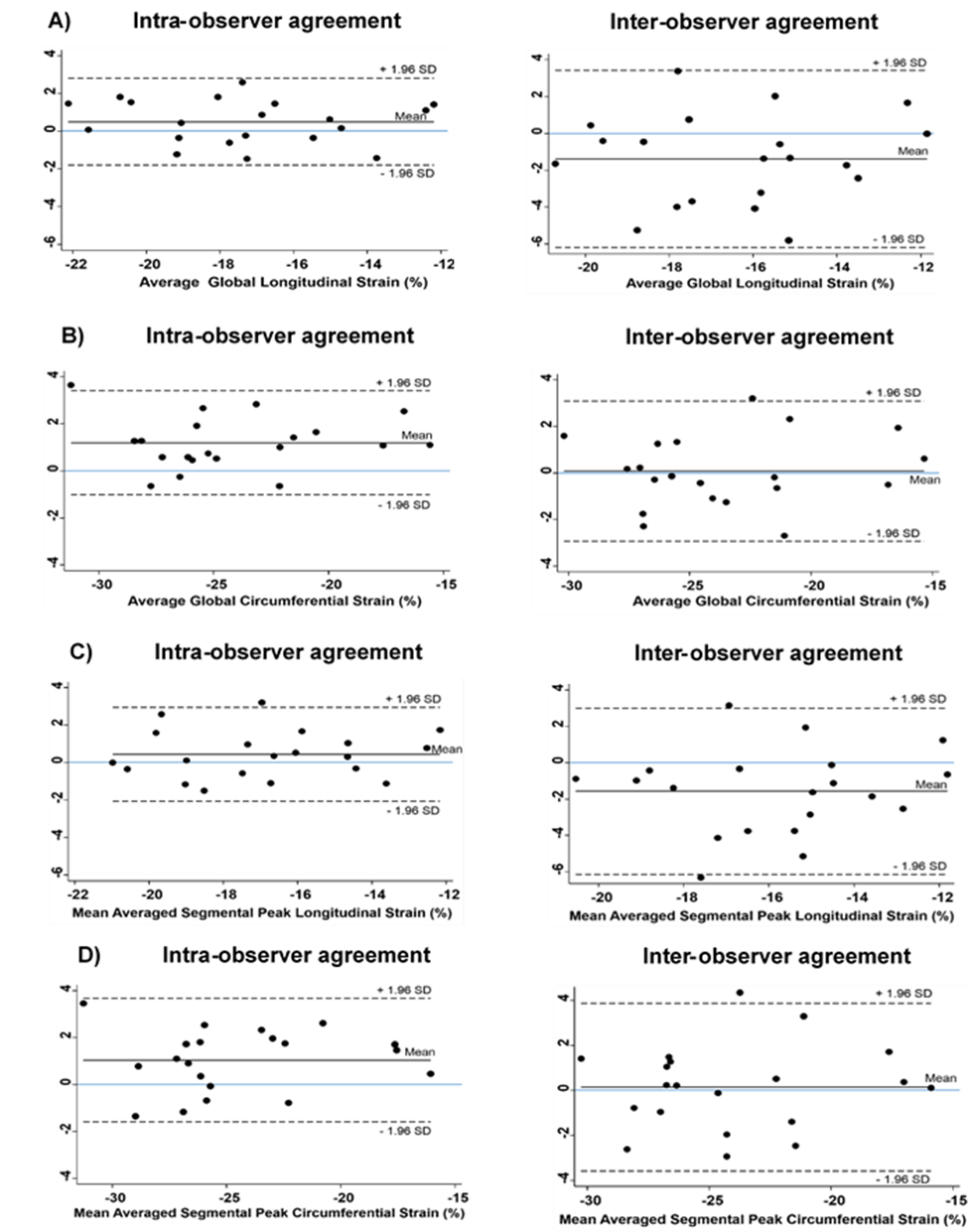


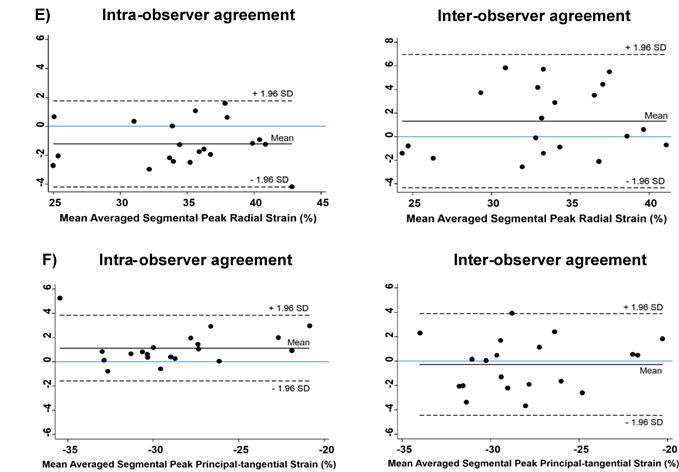


Rotational indices (3D-STE) **
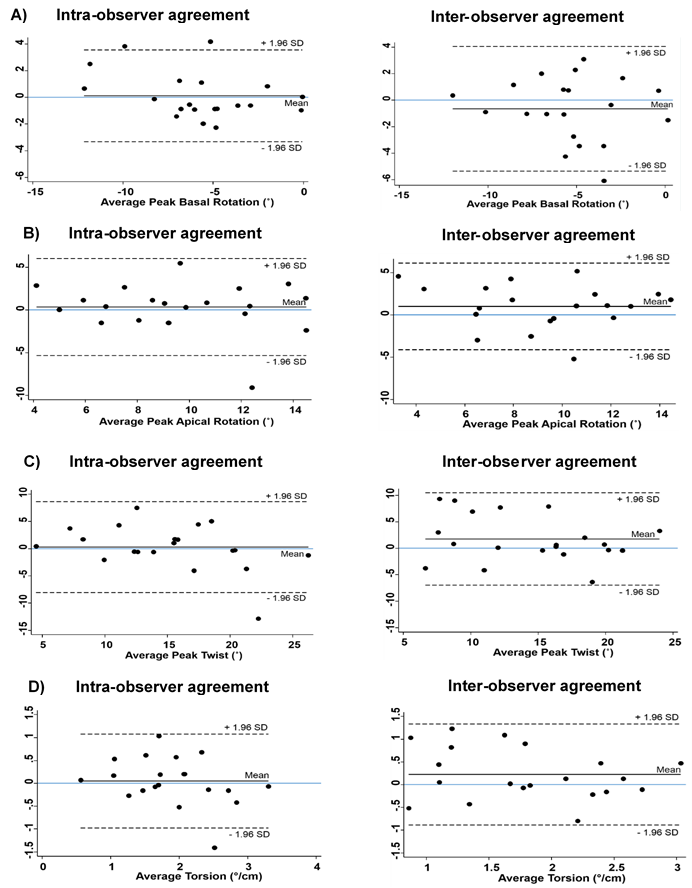
**

Dyssynchrony indices (SDIs; 3D-STE)

**
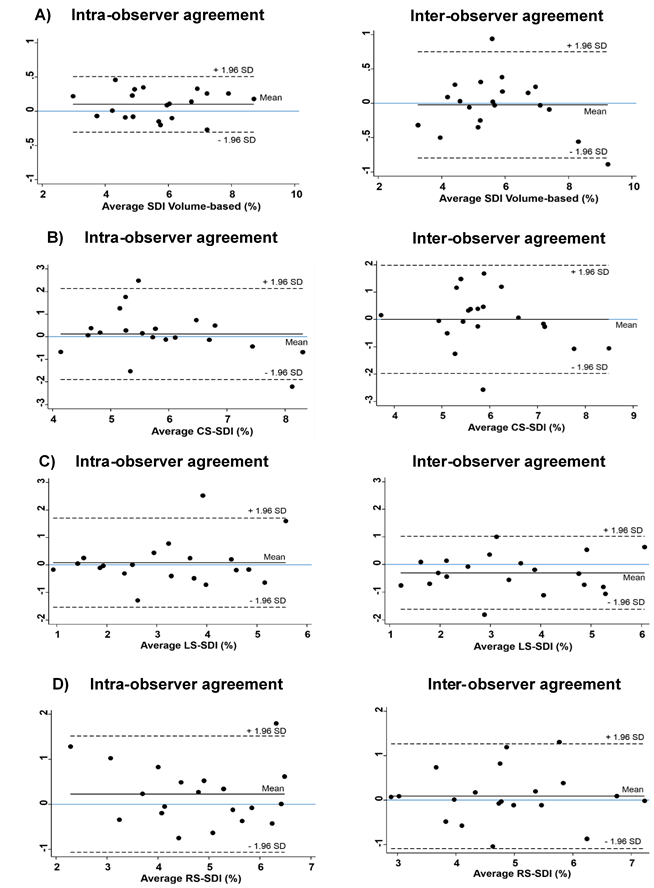
**

**
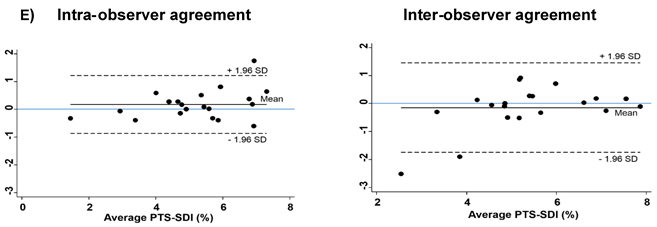
**

Dyssynchrony indices (Di; 3D-STE)


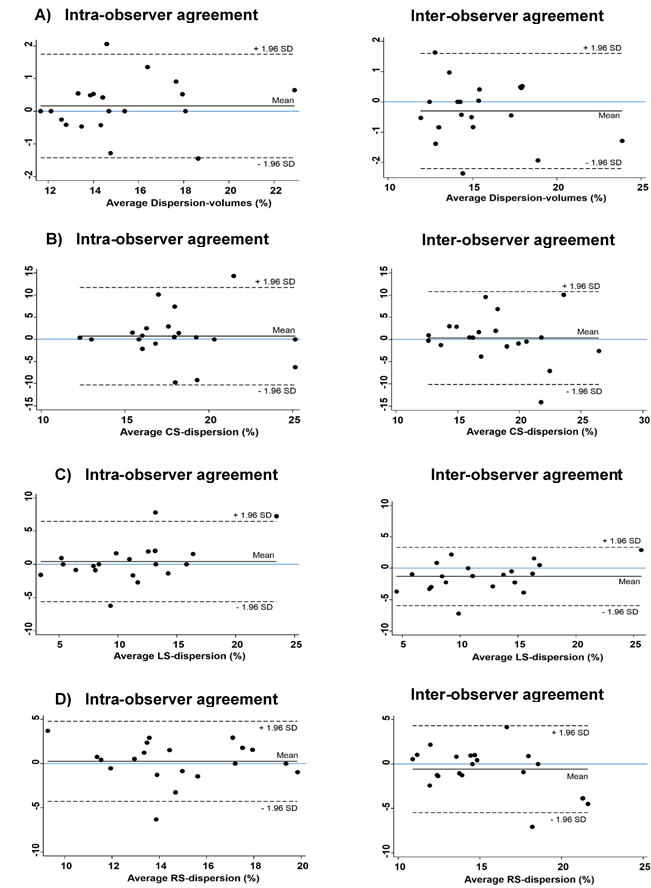


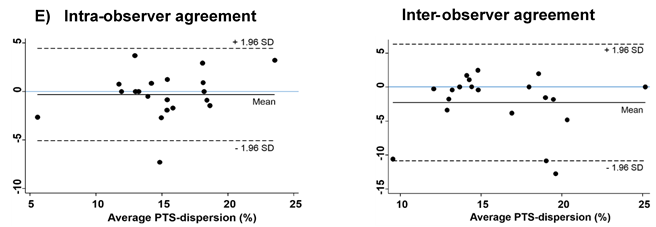


Volumetric indices (3D-STE)

**
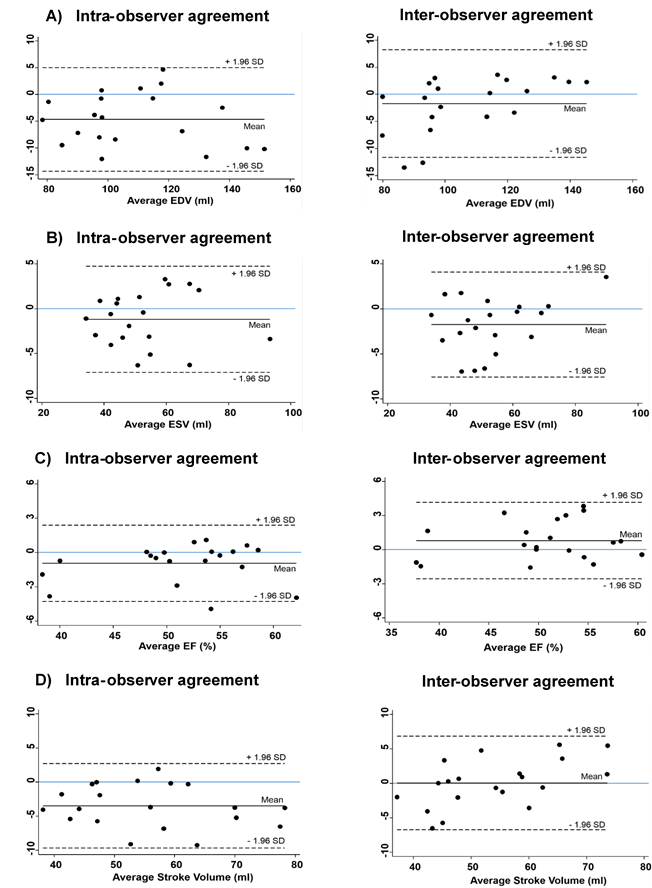
**

**
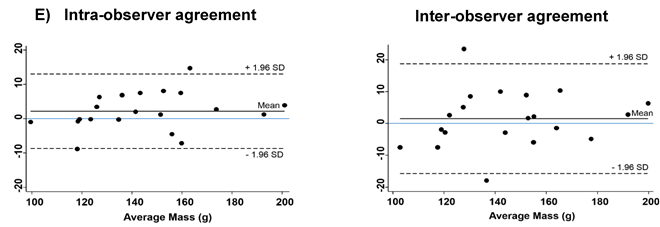
**

***Conventional echocardiographic measures***

2D measures


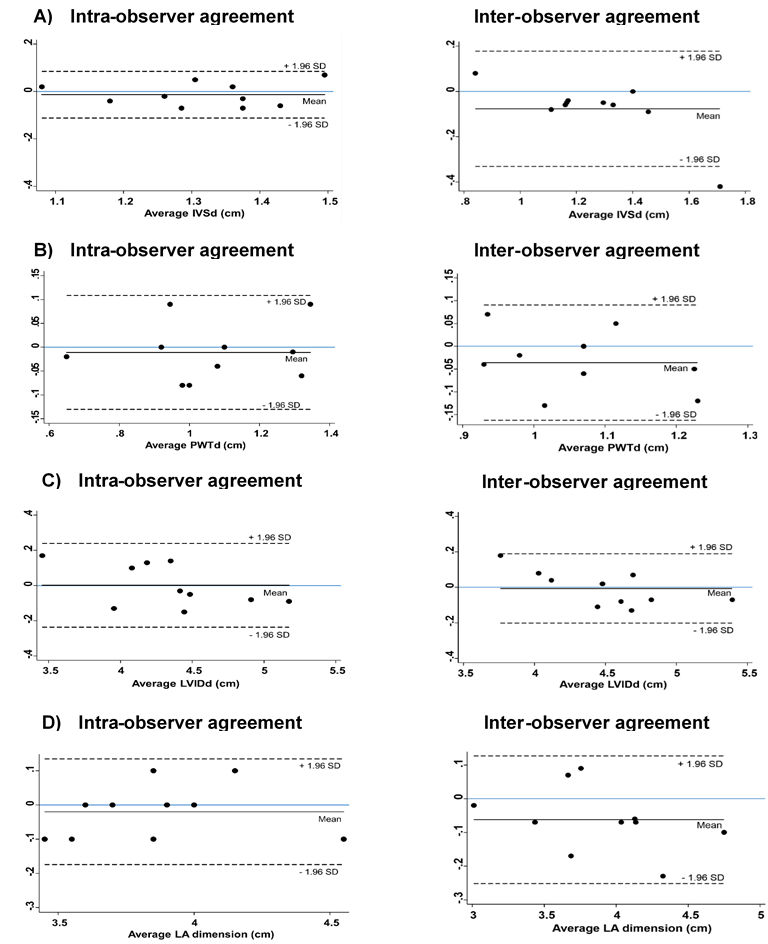


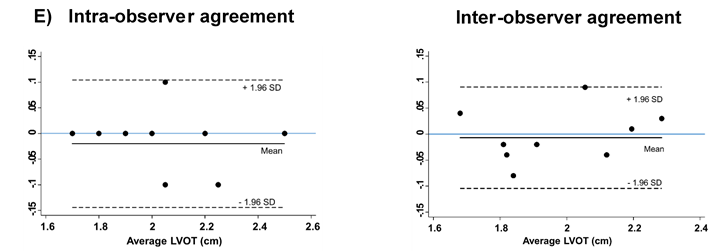


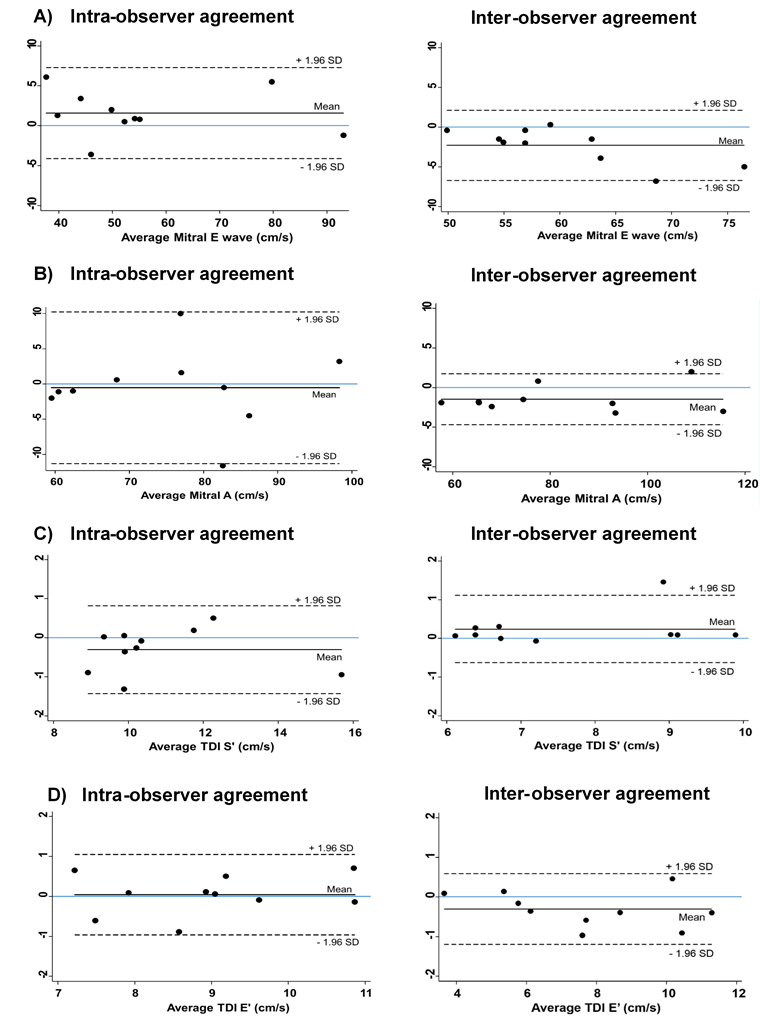
Doppler measures


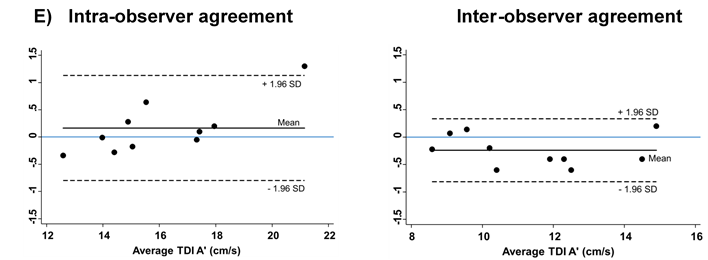


***Vascular measures***

- PWV


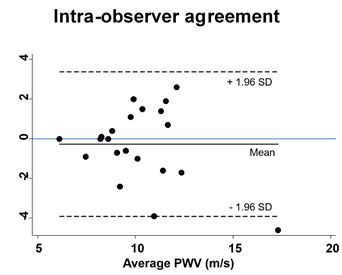


- **
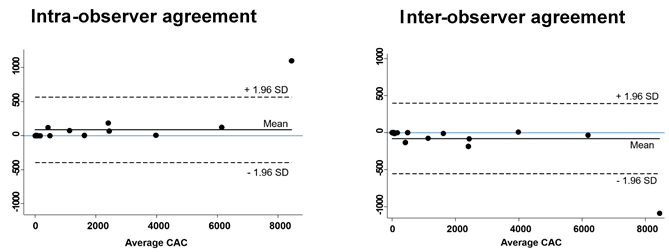
**CACS
-
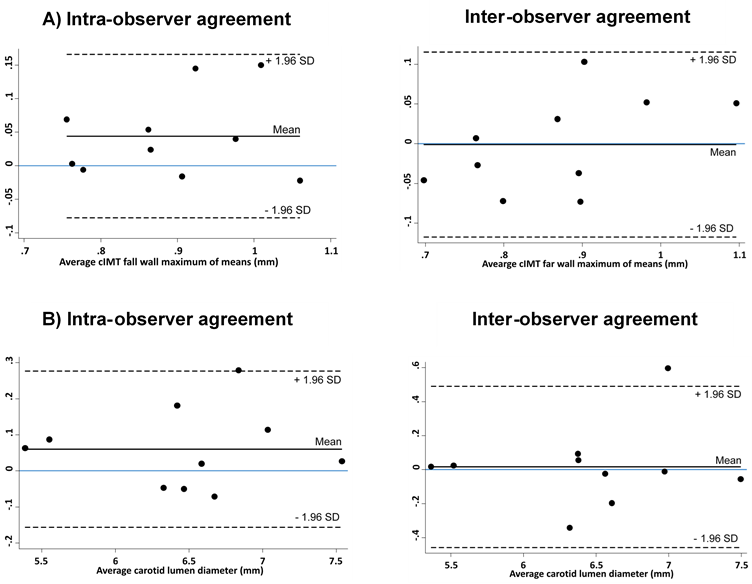
cIMT
- Plaque characteristics


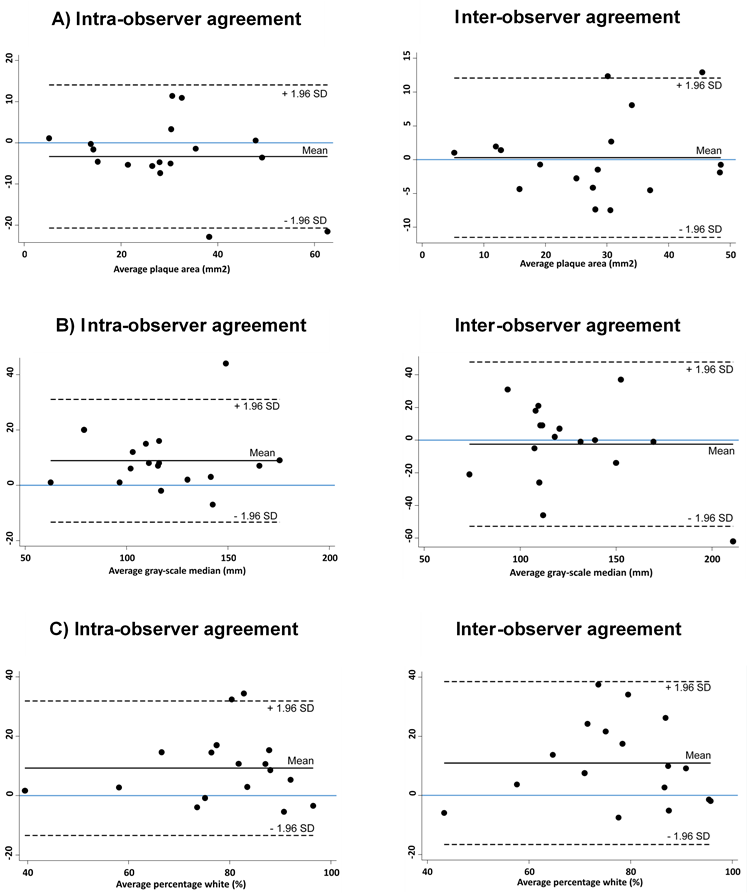

Supplement: Supplementary file 1 [file Data_Sheet_1.docx]
